# A Framework for Assessing the Value of Investments in Nonclinical Prevention

**DOI:** 10.5888/pcd12.150363

**Published:** 2015-12-10

**Authors:** George Miller, Charles Roehrig, Pamela Russo

**Affiliations:** Author Affiliations: Charles Roehrig, Altarum Institute Center for Sustainable Health Spending, Ann Arbor, Michigan; Pamela Russo, Robert Wood Johnson Foundation, Princeton, New Jersey.

## Abstract

We present a high-level framework to show the process by which an investment in primary prevention produces value. We define primary prevention broadly to include investments in any of the determinants of health. Although it builds on previously developed frameworks, ours incorporates several additional features. It distinguishes direct and upstream determinants of health, a distinction that can help identify, describe, and track the impact of a policy or program on health and health care costs. It recognizes multiple dimensions of value, including the need to establish the nonhealth value of investments whose objectives are not limited to improvements in health (and whose costs should not be attributed solely to the health benefits). Finally, it emphasizes the need to describe value from the perspectives of the multiple stakeholders that can influence such investments.

## Background

Despite having the highest per capita health spending in the world, the United States lags behind other developed nations across many health indicators, resulting in a growing interest in what defines and produces health. Some have argued that the small share of US health expenditures devoted to disease prevention — less than 9 % (1) — contributes to this paradox. However, research suggests that many benefits from the nation’s high level of health care spending are undermined by a low level of investment in social services (2), such as support for senior adults, disability and sickness benefits, employment programs, unemployment benefits, housing programs, and other policy initiatives. There is also increasing evidence that nonclinical factors such as education and income have a major impact on health.

Some researchers (3,4) focused on understanding the interplay among health determinants (the clinical and nonclinical factors that affect people’s health) and calculated a range of estimates of the relative contribution of each of these determinants (5). The goal of such research is to design effective interventions that value health for all people and that address not only the direct determinants of health, such as medical care and health behaviors, but also the structural, institutional, and societal circumstances that may cause persistent health disparities. A better understanding of what influences health outcomes should point the way toward more effective use of limited resources to improve population health. Numerous researchers (4,6) and organizations, such as the World Health Organization (WHO) (7), the Centers for Disease Control and Prevention (8), and the Institute of Medicine (9), have proposed frameworks to explain these complex relationships. We propose a new, high-level framework to represent the process by which an investment in primary prevention produces value in terms of its impact on health, costs, and other outcomes of interest to various stakeholder groups. Primary prevention in this context is defined broadly to include investments in the nonclinical determinants of health, often at a community level, and our focus is on such nonclinical primary prevention investments. Our framework builds on those developed previously but extends this work to 1) distinguish direct and upstream determinants of health, 2) recognize multiple dimensions of value (including the nonhealth benefits of some investments whose costs should not be allocated solely to health benefits), and 3) emphasize that value is perceived differently by different stakeholders.

## The Framework

The Figure illustrates our framework. A primary prevention intervention via an investment in one or more of the determinants of health has eventual impact in the form of changes in the health of the affected population, changes in health care costs, and other (nonhealth) effects, as well as changes in expenditures associated with the intervention itself. These effects are viewed differently by different stakeholder groups. 

**Figure:**
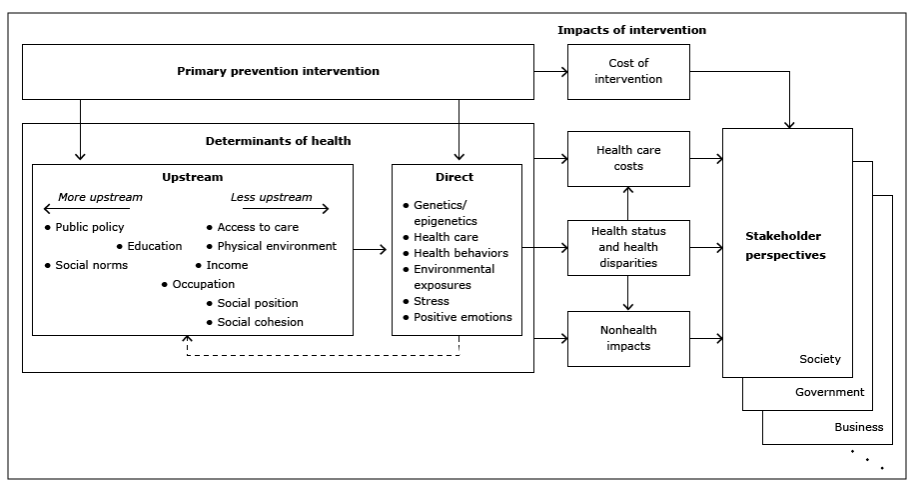
High-level framework. A primary prevention intervention modifies health determinants to affect health, costs, and other factors. These effects are viewed differently from the perspectives of various stakeholder groups that can influence investment in the intervention.

## Primary Prevention Intervention

We consider any investment in a health determinant other than medical care to be a nonclinical primary prevention intervention (10). These investments can range from interventions in upstream determinants, such as improvements to education (eg, providing preschool scholarships) or the built environment (eg, improving access to exercise opportunities with more parks), to more direct interventions, such as encouraging healthy behaviors (eg, banning smoking in public places) or improving water or air quality (eg, more stringent control of automotive emissions). There are many examples of such interventions and evidence for the effectiveness of each (11,12).

## Determinants of Health

Health determinants are commonly considered to be genetics, health behaviors, social and physical environments, and medical care (13). These factors combine to produce individual and population health; any health-improving intervention must address one or more of these determinants. Nonclinical primary prevention interventions — those not delivered in a clinical setting — typically improve health by reducing the occurrence of health problems or improving general well-being. Such interventions may also improve outcomes from existing health problems, as when better air quality reduces asthma attacks, which is an additional benefit beyond primary prevention. These interventions are often referred to as interventions in the social determinants of health (SDH), which include interventions in education, income, employment, social position, and prevailing discriminatory attitudes and practices. Such interventions therefore contain elements of nonclinical primary prevention — nonclinical interventions that improve health. For example, it has been argued that reducing class sizes in US primary schools leads to improved children’s health and therefore meets the definition of nonclinical primary prevention (14). Note that SDHs can operate differently and have differing relative importance depending on the population of interest.

Our framework incorporates additional structuring of the determinants. There is a smaller set (which we call direct determinants) through which all others (upstream determinants) operate (15). The link between direct determinants and health is straightforward. The health impact of upstream determinants can best be understood through their linkage to the direct determinants.

We define the direct determinants of health as those that can have an immediate biological impact on health; the direct determinants, taken together, fully explain variations in health. Upstream determinants affect health only through their impact on direct determinants. Direct determinants are similar to downstream determinants of health (3) and intermediary determinants (7). Although some researchers are reluctant to employ the term “direct” determinants because it could tempt policymakers into ignoring upstream interventions, the most efficient way to impact direct determinants is often through an intervention on upstream determinants.

We identify 3 of the 5 commonly cited determinants of health (13) as direct: medical care, genetics (including epigenetics to reflect the fact that gene expressions can be affected by health behaviors and environmental exposures), and health behaviors. Each has a direct biological pathway to health. The remaining 2 determinants, social environments and physical environments, include both direct and upstream components. We use the term “environmental exposures” to represent the direct components and add it as a fourth direct determinant. It includes exposures to environmental toxins (eg, air and water pollution, lead paint, asbestos), high-violence neighborhoods, and unsafe surroundings (eg, poorly lit streets, unfenced swimming pools).

We add stress as a fifth direct determinant, because it is not represented in the other 4 determinants. (We view stress as influenced by rather than as a characteristic of the social environment. Thus, we do not consider it an environmental exposure.) Stress may result from concerns about such factors as finances, personal safety, job loss, divorce, or social standing. In a survey of the literature, Thoits (16) concluded that “differential exposure to stressful experiences is a primary way that gender, racial-ethnic, marital status, and social class inequalities in physical and mental health are produced.”

In addition to the negative emotions associated with stress, there are positive emotions, such as feelings of self-worth, that can also affect health (17,18). We have created a sixth direct determinant, “positive emotions,” to cover these effects. Stress and positive emotions can affect both mental and physical health.

The remaining characteristics of the social and physical environments constitute the upstream determinants. We have described these in more detail in our framework, based in part on the WHO’s framework (7). The relative positioning of these characteristics (Figure) represents the extent to which each has an immediate and straightforward impact on the direct determinants, with those that are further upstream listed farther to the left. The dashed arrow from the direct determinants to the upstream determinants in the Figure is intended to show that determinants affect each other via complex feedback and other interactions among the determinants that are not shown explicitly. For example, education affects income, income affects access to care, and improved medical care can affect the ability to pursue education and income.

Incorporating direct determinants in our framework contributes toward identifying, describing, and tracking the impact of a policy or program on health and health care costs in the following ways:

- Providing a structure to enable thinking about mechanisms that link a change in an upstream determinant of health (such as income) to health outcomes, even where existing research results do not allow making such direct links quantitatively.
- Providing a mechanism to help convince stakeholders of the validity of a study linking a policy or program to health outcomes and costs. Identification of likely causal mechanisms should help to explain the health-related effects of modifying an upstream determinant of health (such as education) that has no apparent direct relation to health and health care costs.
- Helping to reduce the effort required to assess the impact of each of many alternative upstream policies and programs (such as those designed to influence diet) on health outcomes and costs, because the effects of the direct determinant often need to be assessed only once.
- Allowing partitioning of the assessment of the impact of a policy or program into 2 analysis components: 1) measuring the impact of the policy or program on the direct determinants, and 2) measuring the impact of the direct determinants on health outcomes and costs. If researchers have already identified the latter impact, only the former needs to be assessed to identify the health and cost effects of a given policy or program. Muennig and Woolf (14) provide an example of such partitioning.
- Encouraging investors to target interventions to characteristics of a given upstream determinant of health. Growing evidence about how an upstream determinant acts through the direct determinants can be used to tailor policies and programs to address those causes. For example, better understanding of the role of education in health could help identify improvements to the educational system that would have the greatest impact.
- Helping drive refinement of metrics associated with each of the direct determinants, such as establishing how best to measure stress.
- Providing leading indicators for near-term, post-implementation tracking of the impact of a policy or program on health outcomes and costs. Because the impact on direct determinants is often more immediate and measurable than the impact on health, tracking changes in the direct determinants associated with a policy or program (such as the impact of a tobacco tax on smoking prevalence) can provide early evidence about program effectiveness.

Furthermore, if research led to identification of a small number of direct determinants that provided most of the health outcome and cost benefits associated with investments in SDH, policy makers could focus on investments with the greatest impact on those determinants. Lantz et al (19) provide a seminal example of research on this topic.

## Impacts of Intervention

We characterize 4 types of impacts that can result from a change in one or more of the determinants of health.

**Health status and health disparities.** Any intervention that directly or indirectly affects the direct determinants of health will also have an impact on the health of the affected population. We show the association of these effects with the direct determinants via the arrow that connects the Direct Determinants box to the Health and Health Disparities box (Figure). These might include impacts on health disparities, as when an intervention targets a disadvantaged population (eg, via improvements to public housing). The effects will occur over time, sometimes with (possibly long) delays (eg, improvements to early education can have health impacts much later in the lives of the affected children). The effects can also be intergenerational (such as with smoking cessation efforts that target pregnant women).

**Health care costs.** Interventions can also have an impact on health care costs. Such impact occurs directly when the intervention is a change in medical care itself, such as introduction of an immunization program, but is likely most significant as a result of changes in the health of the population. Because these health effects occur over time, so do the resultant changes in health care costs, which can have similar delays.

**Nonhealth impact.** Many investments in the determinants of health have purposes other than, or in addition to, improvements in health. For example, job training programs are designed to improve employment opportunities and income of the participants but may also have positive health benefits. Although the primary focus of our framework is to highlight the impact of interventions on health outcomes and health care costs, comparing all the costs of such an investment with only the health benefits that accrue will understate the overall cost-effectiveness of the investment. We include nonhealth impacts of interventions in the framework for this reason. Major types of nonhealth impact include effects on income (and its secondary effects, such as improvements to the economy and increased tax revenues) and on other aspects of nonhealth community well-being — wealth, education, employment, safety, transportation, housing, worksites, food, and recreational spaces (9). Identifying all health and nonhealth benefits of an upstream intervention and appropriately allocating investment costs between them is an important research topic.

**Cost of intervention.** Most interventions have direct costs (such as the cost of hiring additional teachers to improve high school graduation rates [14]) that are combined with other costs or cost offsets (such as revenue from increased cigarette taxes) and compared with the benefits they produce to assess whether they provide good value. The framework provides for capturing these direct costs.

## Stakeholder Perspectives

The impact of a primary prevention intervention is represented in the various categories of costs and benefits that are of interest to the various stakeholders who can influence such investments. These stakeholder perspectives are represented by the boxes on the right side of the framework (Figure). The perspectives convert the detailed effects of a given intervention to metrics that are relevant to each stakeholder group. To ensure that analyses using the framework produce information of value to these various stakeholders (eg, elected and unelected government officials, public health representatives, health care providers, insurers, employers) as well as society as a whole (20), these metrics must capture data on the various benefits and costs of an investment that are most compelling to each group. For example, the health impact of an intervention could be expressed as a change in health-adjusted life expectancy for some stakeholders (21), but the impact of the intervention on absenteeism or presenteeism might be a more meaningful metric for employers. Similarly, an intervention that reduces spending on health care will have a different financial impact for the federal government, state governments, private insurers, individuals, and society.

## Conclusion

The framework described here is designed to help researchers, public health officials, policymakers, and others improve their understanding of the value of an investment in the determinants of health, the mechanisms by which this value is achieved, and the ways in which this value is perceived by the stakeholder groups affected by the investment. Our ongoing research includes development and application of quantitative methods to apply the framework to assessing the value of investments in nonclinical primary prevention from the perspectives of various stakeholders.
